# Factors associated with provision of smoking cessation support to pregnant women – a cross-sectional survey of midwives in New South Wales, Australia

**DOI:** 10.1186/s12884-020-02912-0

**Published:** 2020-04-15

**Authors:** Megan E. Passey, Jo M. Longman, Catherine Adams, Jennifer J. Johnston, Jessica Simms, Margaret Rolfe

**Affiliations:** 1 The University of Sydney, University Centre for Rural Health, PO Box 3074, Lismore, NSW 2480 Australia; 2 Northern New South Wales Local Health District, Locked Mail Bag 11, Lismore, NSW 2480 Australia

**Keywords:** Smoking cessation, Antenatal care, Theoretical domains framework, Health professional behaviour

## Abstract

**Background:**

Smoking is the most important preventable cause of adverse pregnancy outcomes, but provision of smoking cessation support (SCS) to pregnant women is poor. We examined the association between midwives’ implementation of SCS (5As – Ask, Advise, Assess, Assist, Arrange follow-up) and reported barriers/enablers to implementation.

**Methods:**

On-line anonymous survey of midwives providing antenatal care in New South Wales (NSW), Australia, assessing provision of the 5As and barriers/enablers to their implementation, using the Theoretical Domains Framework (TDF). Factor analyses identified constructs underlying the 5As; and barriers/enablers. Multivariate general linear models examined relationships between the barrier/enabler factors and the 5As factors.

**Results:**

Of 750 midwives invited, 150 (20%) participated. Respondents more commonly reported Asking and Assessing than Advising, Assisting, or Arranging follow-up (e.g. 77% always Ask smoking status; 17% always Arrange follow-up). Three 5As factors were identified– ‘*Helping’*, ‘*Assessing quitting*’ and ‘*Assessing dependence*’. Responses to barrier/enabler items showed greater knowledge, skills, intentions, and confidence with Assessment than Assisting; endorsement for SCS as a priority and part of midwives’ professional role; and gaps in training and organisational support for SCS. Nine barrier/enabler factors were identified. Of these, the factors of ‘*Capability’* (knowledge, skills, confidence); ‘*Work Environment*’ (service has resources, capacity, champions and values SCS) and ‘*Personal priority*’ (part of role and a priority) predicted ‘*Helping’*.

**Conclusion:**

The TDF enabled systematic identification of barriers to providing SCS, and the multivariate models identified key contributors to poor implementation. Combined with qualitative data, these results have been mapped to intervention components to develop a comprehensive intervention to improve SCS.

## Background

Smoking is the single most important preventable cause of adverse infant outcomes including stillbirth, preterm birth, low birth weight, asthma, childhood respiratory infections and adult cardiovascular disease [1–4]. These harms are significantly reduced if women stop smoking during pregnancy [2]. Many pregnant women are highly motivated to quit smoking, but often face significant challenges in doing so, including lack of support from health professionals [5]. Psychosocial interventions to support pregnant women to quit are effective [2].

In Australia, clinical guidelines recommend routine and repeated assessment of smoking status for all pregnant women with ongoing support for quitting and staying quit, using the evidence-based 5As framework (Ask, Advise, Assess, Assist and Arrange follow-up) [6]. This means that, at every antenatal visit, the woman should be *Asked* her smoking status. If she is a current smoker, or has recently quit smoking, the remaining As are provided, as relevant. The woman should be *Advised* that the best thing she can do for her baby is to stop smoking, with discussion of the benefits of doing so. Her nicotine dependence and willingness to quit should be *Assessed*. She should then be *Assisted,* for example, encouraging her to stop smoking using motivational interviewing techniques, and providing resources and referral, as appropriate. Finally, the clinician should *Arrange follow-up* to provide additional support and monitor progress [6]. The 5As framework is effective, considered to be best practice and widely adopted in policy and practice both in Australia and internationally [7, 8]. However, implementation of the 5As continues to be poor with many women not provided with assistance for quitting [9, 10]. In New South Wales (NSW), only 46% of women who smoked in pregnancy recalled being told about quitting programs [11]. Qualitative research has confirmed gaps in care, especially with Assisting and Arranging follow-up [12]. The failure to deliver smoking cessation support (SCS) is a critical missed opportunity to support cessation.

Changing the practice of health care providers requires the use of a comprehensive theory-driven and evidence-based approach which addresses specific behaviours in context [13]. One such approach, the ‘Behaviour Change Wheel’ (BCW), has demonstrated effectiveness in changing healthcare delivery [14]. The BCW approach starts with identification of the target behaviour for change, then development of a clear understanding of the barriers to its implementation. The BCW categorises barriers to implementing behaviours under three main constructs: Capability (Psychological or physical ability to enact the behaviour, e.g. having the required skills and knowledge); Opportunity (Physical and social environment that enables the behaviour, e.g. having the required resources to undertake the behaviour); and Motivation (Reflective and automatic mechanisms that activate or inhibit the behaviour, e.g. perceiving the behaviour as an important part of their role) [14]. Once the barriers to the behaviour have been identified and understood, the intervention is developed by mapping the identified barriers to intervention functions and behaviour change techniques [14]. Further detail on this process can be found in ‘*The Behaviour Change Wheel: A Guide to Designing Interventions*’. [14].

The Theoretical Domains Framework (TDF) is a validated, comprehensive framework for identifying barriers and enablers to implementation [15] and can be used in the initial ‘diagnostic’ phase of the BCW process. The TDF was validated with 37 experts in behaviour change theory, using closed and open sort tasks with testing of the extent of replication by discriminant content validation and fuzzy cluster analysis [15]. It covers 14 domains of theoretical constructs (Knowledge, Skills, Memory, Attention and Decision Processes, Beliefs about Capabilities, Beliefs about Consequences, Optimism, Intentions, Goals, Emotion, Professional Role and Identity, Social Influences, Environmental Context and Resources, Behavioural Regulation, and Reinforcement) [15]. The TDF has previously been used to identify barriers to implementation, including barriers to providing SCS to women [16], with subsequent development of an effective intervention [17].

The **objective** of this paper is to examine the association between midwives’ self-reported implementation of the 5As and reported barriers and enablers to their implementation, in order to identify critical areas for interventions designed to increase provision of SCS.

## Methods

A cross-sectional on-line anonymous survey of midwives providing antenatal care in the public health system in NSW, Australia, was undertaken over 4 weeks in late 2016. Ethical approval was provided by the Hunter New England Human Research Ethics Committee (Ref. 14/06/18/5.04).

### Questionnaire development

An initial draft questionnaire was developed based on the 14 domains of the TDF [15] and a review of literature and other instruments using the TDF [14, 16, 18]. The questionnaire also included items assessing provision of each of the 5As. These items were adapted from those used by Jordan et al [19], in order to reflect the NSW Guidelines for smoking cessation support during pregnancy [6] and asked about how often each of the 5As was undertaken. These items were specifically designed to provide most emphasis on Assisting as this was identified as a critical gap in the literature. Items covering professional characteristics and personal smoking status were also included (see below under questionnaire content for details).

The questionnaire was assessed for face and content validity by five midwives with extensive experience in antenatal care and by a behavioural scientist with expertise in the use of the TDF. Face validity assesses whether an instrument appears to assess the desired qualities, and content validity assesses whether it covers all the important domains – both are subjective assessments [20]. The reviewers confirmed the comprehensiveness, acceptability and relevance of the items, with minor suggestions for improvements. In response to these suggestions, further refinements were made, with minor changes to wording for clarity, the addition of two items, reordering to reflect order of activities in antenatal care, and the reallocation of two items to different domains (one from ‘goals’ to ‘beliefs about capabilities’ and one from ‘goals’ to ‘environmental context and resources’). The response format was well received and considered easy to follow and use. It was recommended to keep it as short as possible while covering key constructs, to maximise completion by busy midwives. The revised questionnaire was again reviewed by two of the five midwives, with final minor adjustments made to the ordering of items.

### Questionnaire content

The final questionnaire contained 59 items, covering professional characteristics (five items), perceived barriers and enablers to providing the 5As (41 items), reported provision of the 5As (12 items) and participants’ smoking status (one item).

Professional characteristics included years of midwifery experience, all models of antenatal care the respondent was currently working in, main model currently working in, annual number of births in their service and which Local Health District (LHD) they worked in.

Barriers and enablers covered all 14 TDF domains. A 5-point Likert scale response format was used, from strongly agree (score 1) to strongly disagree (score 5).

Respondents were asked to indicate the frequency with which they undertook each of the 5As. A 5-point Likert scale response format was used, from always (score 1) to never (score 5).

Smoking status response options were: I smoke every day; I smoke occasionally, but not every day; I’m an ex-smoker, I never smoke now; I have never smoked.

The questionnaire was administered in REDCap, a secure application for managing online surveys and databases. The questionnaire took less than 15 min to complete and was anonymous, thus ensuring confidentiality for participants. It is available in Additional file 1.

### Participants and recruitment

As we were interested in developing an intervention to improve implementation of the 5As during pregnancy across NSW, we wanted to include all midwives providing antenatal care in the NSW Public Health system, rather than focus on just one area or facility. The NSW Public Health system is administered through 15 LHDs. All LHDs were invited to participate, and participants were eligible if they were a midwife currently providing antenatal care in any of these LHDs. Eligible midwives were invited to participate by Clinical Midwifery Consultants (CMCs) in 14 of the 15 NSW LHDs, with one LHD not participating. CMCs are senior midwives whose role includes development of policy and strategy to ensure high quality care, including support for research to improve care. The project had been discussed and endorsed by the CMC network, with agreement to distribute the online link to eligible midwives. The CMCs were sent an email script to invite midwives and asked to distribute to all eligible midwives in their district. Three reminders were sent to CMCs and the survey was open for 4 weeks. An incentive of AUD300 for use for professional development was offered to the LHD with the greatest proportion of eligible midwives participating (CMCs provided data on the number of eligible midwives each had invited).

### Analysis

Data were analysed in SPSS (version 22) by a biostatistician (MR). Descriptive summary statistics were generated for all items.

Prior to assessing the associations between provision of the 5As and the barriers and enablers to their implementation, we undertook two separate factor analyses – one to identify the constructs underlying the provision of the 5As, and one to identify constructs underlying the barriers/enablers. This was done in order to reduce the number of tests required to assess the associations between the provision of the 5As and the barriers/enablers, thus reducing the risk of type 1 error. The factor analyses also facilitated assessment of the construct validity of the instrument.

Anti-imaging correlation matrix, Bartlett’s test of sphericity and measure of sampling adequacy were used to identify the appropriateness of using factor analysis. Principal axis factoring (as we expected latent constructs) using the correlation matrix with both oblique (promax) and orthogonal (varimax) rotations were investigated. Factors with eigenvalues greater than 1 were extracted. For the oblique rotation, both the factor pattern matrix and the factor structure matrix were assessed. Negatively worded items were reverse coded. For the barriers/enablers analysis, due to paucity (none or one) of responses in the extreme ‘Strongly disagree’ category for 20 of the 41 items, the responses ‘disagree’ and ‘strongly disagree’ were combined, resulting in a four category Likert scale. The subsequent factor analysis using these responses on 41 items exhibited convergence problems, which were resolved by the exclusion of a single item. The excluded item ‘I have good knowledge of the harms of smoking’ had 98.5% responses with either ‘strongly agree’ or ‘agree’ providing minimal discrimination. Cronbach’s alpha (> 0.7), item-to-item (> 0.3) and item to total (> 0.5) correlations were used to assess adequate reliability or internal consistency of resultant factors. For all factors, variable items were coded such that higher scores indicated more frequent or optimal responses with multi-item factor composite scores being calculated as the mean of the items.

Associations between the resultant 5As factors and the barrier/enabler factors were then assessed using general linear model techniques with each of the 5As factors as dependent variables, and the barrier/enabler factors (continuous variables), midwives’ smoking status (‘never smoked’, ‘ever smoked’ (current or past smokers)); and midwifery years of experience (‘0–5 years’, ‘6–10 years’, ‘11–15 years’ and ≥ 16 years) as explanatory categorical variables. The interaction between smoking status and years of experience was also investigated. Univariate analyses were conducted prior to developing multivariate models. A backward stepwise process was used to determine which barrier/enabler factors contributed significantly to each of the 5A factors. All barrier/enabler factors as well as midwife smoking status and years of experience (as potential confounders) were included, then the least significant one excluded in a stepwise manner until all retained explanatory variables were significant (*p* < 0.05).

## Results

### Respondent characteristics

Of 750 midwives invited, 150 completed the questionnaire, giving a response rate of 20.0%. Midwives reported a broad range of models of antenatal care, service size and years of service (Table 1). The majority (63%) reported never smoking and one third were ex-smokers, with a small number reporting current smoking.

**Table 1:** Respondent and service characteristics

Characteristic	n	%
Model of antenatal care MOSTLY worked in		
Midwife and GP Shared Care	72	48
Midwifery group practice	30	20
Obstetric-led	26	17
Team midwifery	13	9
Aboriginal Maternal and Infant Health Service	8	5
Publically funded homebirth	1	1
Size of Service (births per year)		
≤ 500 births	44	29
501–2000 births	36	24
> 2000 births	70	47
Number of years working in midwifery		
0–5 years	32	21
6–10 years	17	11
11–15 years	81	54
> 15 years	20	13
Smoking status		
I smoke every day	3	2
I smoke occasionally, but not every day	3	2
I’m an ex-smoker – I never smoke now	48	32
I have never smoked	95	63
Missing	1	1

### Self-reported provision of 5As

**Fig. 1:**
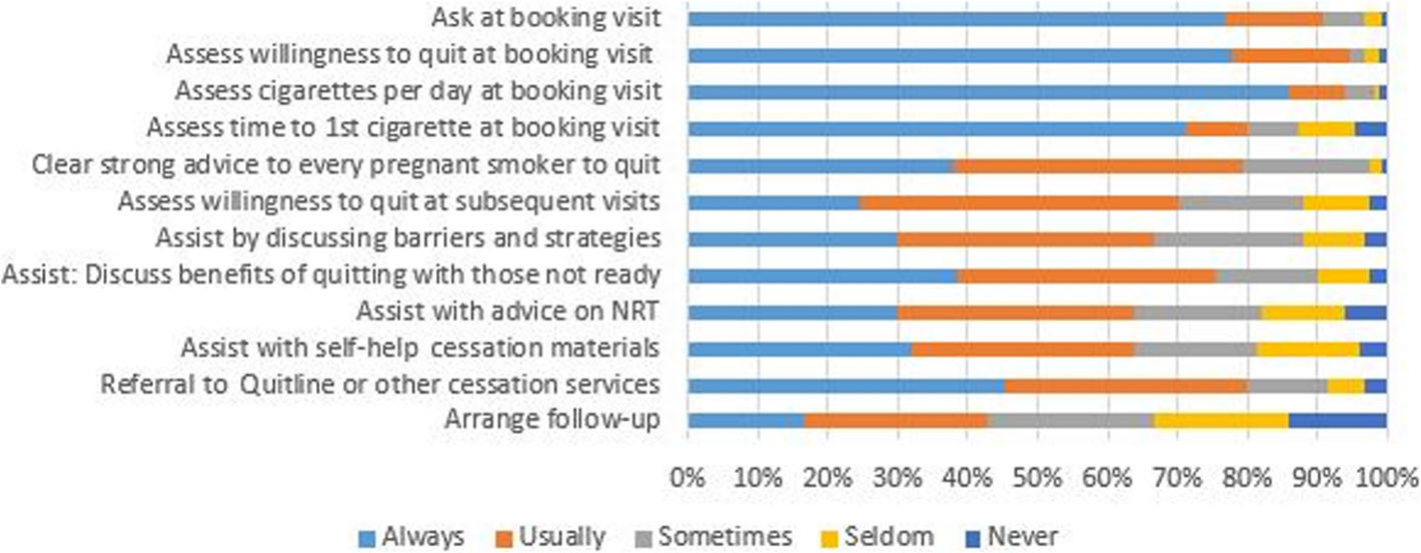
Reported frequency of implementing the 5As

Respondents more commonly reported Asking and Assessing than Advising, Assisting, or Arranging follow-up, although Assessing at subsequent visits was also less common (Fig. 1).

#### Constructs underlying implementation of the 5As

Factor analysis of the 5As responses identified three composite factors, representing three underlying constructs, with eigenvalues> 1, explaining 63.5% of the variance. Factor 1 (named ‘*Helping’*), included the Advise item, the five Assist items and the Arrange follow-up item. Factor 2 (named ‘*Assessing quitting’*) included the Ask item and the two Assess readiness items. Factor 3 (named ‘*Assessing dependence’*) included the two Assess nicotine dependence items. The factors, their loadings from the pattern matrix and Cronbach’s alpha values are shown in Additional file 2.

### Reported barriers and enablers to provision of 5As

Consistent with responses regarding the provision of the 5As, responses to items about barriers and enablers showed greater knowledge, skills, intentions, and confidence with Assessing than with Assisting. Responses indicated endorsement by midwives for supporting smoking cessation as an important priority and part of their professional role. However, responses also indicated considerable gaps in training and organisational support for SCS. Full responses are provided in Additional file 2.

#### Constructs underlying barriers and enablers

**Fig. 2:**
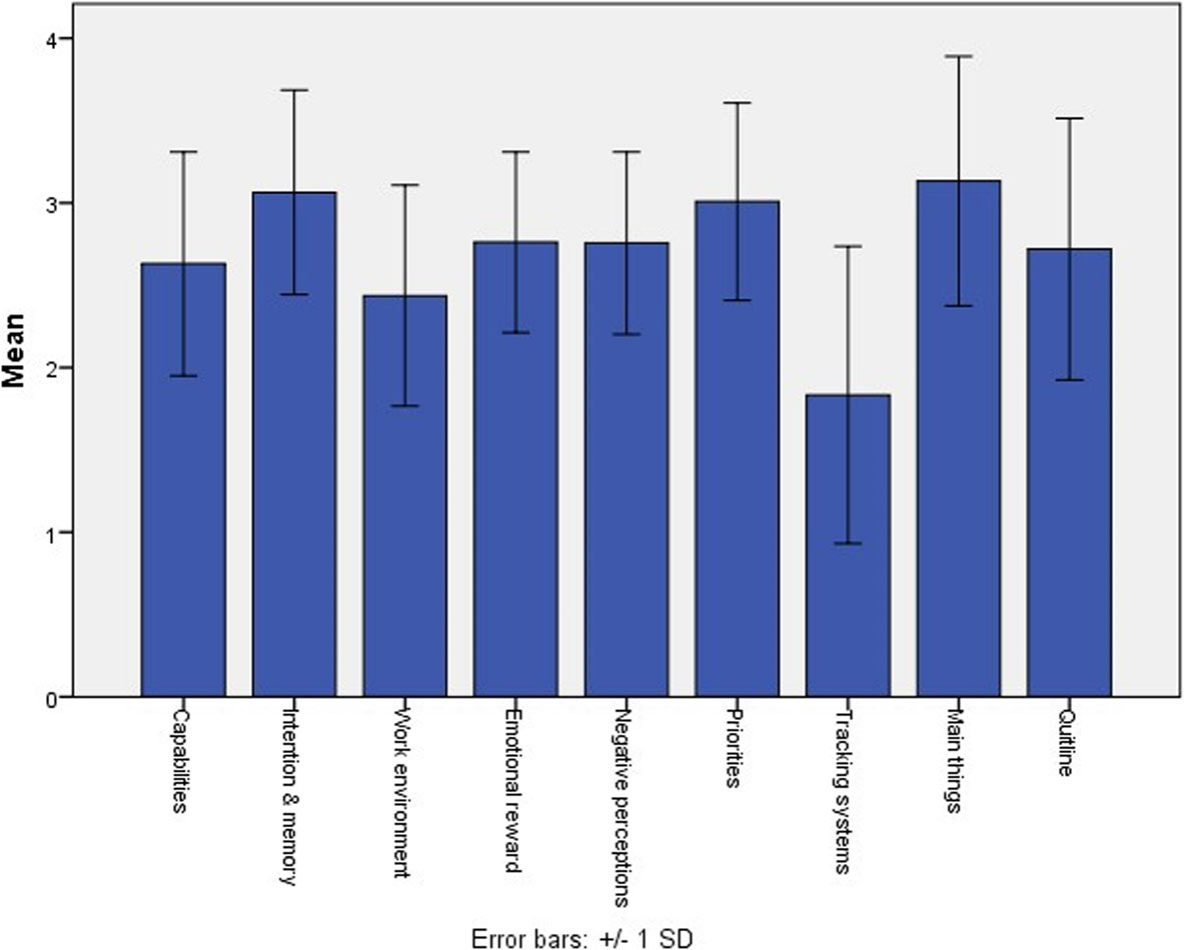
Mean scores for barrier/enabler factors

The factor analysis identified nine factors with eigenvalues> 1 from the 40 barriers/enablers items, explaining 66.5% of the total variance. There were seven composite factors with Cronbach’s alpha statistics > 0.7 confirming internal consistency and two single item factors. The first factor (‘*Capability’*) explained 32% of the variance, and the first four factors explained over 50% of the total variance. The factor structure had clear conceptual coherence. The factors, their loadings from the pattern matrix and Cronbach’s alpha values are shown in Table 2. Mean scores for each factor are shown in Fig. 2.

**Table 2:** Factor categories and loadings for the barriers/enablers of implementation of the 5As

Factor (Cronbach’s alpha)	Item	Loading
Capability (0.927)	I have good knowledge of the use of NRT in pregnancy	.863
	I have good skills in assisting pregnant women who are struggling to quit	.795
	I have good skills in assisting pregnant women with strategies to quit smoking	.776
	I have the skills required to determine and interpret pregnant women’s nicotine dependence	.774
	I know how to provide smoking cessation support in antenatal care to help pregnant women quit	.768
	I have good knowledge of nicotine addiction and the barriers to quitting smoking	.761
	I am confident providing smoking cessation assistance to pregnant women	.751
	I am confident assessing women’s smoking status	.721
	I am confident arranging follow-up support for pregnant smokers	.631
	I’ve had adequate training in assisting pregnant women to quit smoking	.584
	I have good skills in motivating pregnant women who don’t want to quit, to try to quit	.583
	I am familiar with the guidelines for using the 5As for smoking cessation during antenatal care (Ask, Advise, Assess, Assist, Arrange Follow-up)	.497
Intentions and memory (0.826)	I intend to provide smoking cessation support to each pregnant smoker	.878
	I intend to follow up with all smokers about their smoking at later visits (after the booking in visit)	.810
	I always remember to advise women who smoke to quit smoking	.689
	I intend to advise all pregnant smokers to quit	.612
	I always remember to provide smoking cessation support to smoking women at every antenatal visit	.519
Work environment (0.796)	Our service has good pamphlets and resources to support pregnant smokers to quit	.965
	Our service has capacity to provide smoking cessation support for pregnant smokers	.739
	Our service has midwives, obstetricians and/or managers who really champion addressing smoking with our clients	.611
	The clinic I work in values midwives who follow the 5As guidelines	.554
	The team I work with places a high priority on addressing smoking with pregnant women	.457
Emotional reward (0.767)	Helping women quit smoking makes me feel proud of my role	.778
	I get satisfaction from providing smoking cessation support to pregnant women	.591
	I think providing smoking cessation support for pregnant women increases the chances that they’ll quit	.584
	I feel optimistic that providing smoking cessation support helps women quit smoking	.488
	Most women appreciate it when I discuss quitting smoking with them	.361
Negative perceptions (0.720)	I often find talking with pregnant smokers about their smoking makes me feel uncomfortable	.715
	Advising women to quit smoking risks pushing them away from antenatal care	.704
	After the booking in visit, providing smoking cessation support is not as important to me as providing some other aspects of antenatal care	.461
	I don’t have time to provide smoking cessation support in visits after the booking in visit	.453
	The harms of smoking in pregnancy are not as great as the other risks that women face	.426
	Providing smoking cessation support to women is not worth it given the small level of success	.377
Personal priority (0.782)	Providing smoking cessation support for pregnant women is an important part of my role	.752
	Talking with women about quitting smoking is a good use of my time	.528
	I place a high priority on helping women quit smoking	.406
Tracking systems (0.84)	I have systems in place (e.g. a checklist or stickers) to help me keep track of women who smoke and provide ongoing smoking cessation support for them	.936
	Our service has systems in place to help keep track of women who smoke and provide ongoing support for them	.574
Main things	Advising women to quit smoking is one of the main things that can be done to help women have healthy babies	.328
Quitline	Referring women to the Quitline is an effective way of assisting pregnant women to quit	.496

### Association between barriers and self-reported provision of the 5As

Associations between the 5As factors ‘*Helping’* and ‘*Assessing quitting’* and the explanatory variables (nine barrier/enabler factors) and potential confounders (smoking status and years of experience) were assessed using univariate general linear modelling. All associations were statistically significant with two exceptions – the association of ‘*Helping’* with ‘*Main things’* and the association of ‘*Assessing quitting’* with ‘*Quitline*’. Higher scores on each of the barrier/enabler factors were associated with provision of higher levels of ‘*Helping’* and ‘*Assessing quitting’* and ever smokers provided higher levels of support than never smokers. As there was little variability in 5As Factor 3 (‘*Assessing dependence’*) we did not assess associations of explanatory variables with this factor.

The final multivariate models for ‘*Helping’* and ‘*Assessing quitting’* are shown in Table 3. Midwives who more strongly agreed with the items in the ‘*Capability’*, ‘*Work environment’* and ‘*Personal priority’* factors reported higher levels of ‘*Helping’* actions. The important barrier factors for ‘*Assessing quitting’* were somewhat different, with ‘*Intentions and memory’, ‘Work environment’, ‘Negative perceptions’* (disagreeing) being significantly associated. Ever smokers provided higher levels of support than never smokers.

**Table 3:** Multivariate models of the association between ‘*Helping’* and ‘*Assessing quitting’* and the barriers/enablers factors

Adjusted R^2^	‘Helping’				‘Assessing quitting’			
	0.431				0.298			
	F	β	SE	p	F	β	SE	*p*
Model *p* value				< 0.001				< 0.001
Barriers/enablers constructs								
Capability (1)	9.36	0.304	0.099	0.003				
Intentions & memory (2)					9.26	0.263	0.086	0.003
Work environment (3)	16.04	0.379	0.095	< 0.001	4.19	0.159	0.077	0.042
Negative perceptions (5)					11.62	0.313	0.092	0.001
Personal priority (6)	8.09	0.283	0.099	0.005				
Personal characteristics								
Smoking status	6.20			0.014	10.31			0.002

Note: Each of the variables in the final models account for 1 df (error df = 145)

## Discussion

In this paper we have assessed midwives self-reported provision of SCS to pregnant women, explored their perceptions of the barriers and enablers to providing this support and examined the relationship between these barriers and enablers and the support provided. This allowed identification of the most critical barriers and enablers that need to be addressed to improve delivery of effective SCS. Importantly, in developing our questionnaire, we used a comprehensive and validated theoretical framework, the TDF, which will facilitate the process of identifying intervention components using the BCW process [14]. The factor analyses of the two main components of the questionnaire identified three 5As factors and nine barrier/enabler factors with the factors in each analysis explaining approximately two thirds of the variance, all composite factors having high Cronbach’s alpha, and the structures having conceptual coherence, thus providing evidence for the validity of the questionnaire.

Asking and Assessing (especially nicotine dependence) were more commonly performed than Advising, Assisting and Arranging follow-up, and smoking was more commonly addressed at the initial visit than subsequent antenatal visits (Fig. 1). By contrast, other studies have found that Asking smoking status and giving brief Advice to quit smoking were more commonly performed than Assessing, providing Assistance or Arranging follow-up [19, 21–24]. In the case of provision of advice, this difference may result from the wording of our question (‘How often do you give *clear, strong messages urging* every pregnant smoker to quit?’). Most other studies have asked about providing ‘*brief advice’* which may account for the higher positive response. Chang et al audio-recorded 116 interactions at the first obstetric visit and identified that obstetricians Asked about smoking in 98% of visits but only provided Advice to quit in 31% of visits. In only six of the 116 cases was the Advice considered to be ‘*strong’* advice, and in only four cases was it ‘*clear, strong and personalised’*. [25].

The high rate of Assessing nicotine dependence is likely due to the clinical information system used by midwives, which made assessment of nicotine dependence (time to first cigarette and cigarettes/day) mandatory fields for all smokers. The inclusion of these mandatory fields in the clinical information system also explains the lack of variability in response to these questions and highlights the importance of electronic information systems in providing prompts for clinical activity [26].

.The finding that smoking is more frequently addressed at the initial visit than at subsequent visits is consistent with multiple other studies with both midwives and obstetricians [12, 22, 24, 27, 28]. Offering personalised support for cessation repeatedly throughout pregnancy is valuable as it provides a consistent message for women that midwives are concerned about them and their smoking [29, 30].

.The factor analysis of the 5As questions identified three factors, with one main factor (named ‘*Helping’*) including all the Advice, Assist and Arrange follow-up items, with strong factor loadings (all > 0.58). The other two factors, ‘*Assessing Quitting’* and ‘*Assessing Dependence’,* related to assessing women’s behaviour. While making assessments is useful for clinicians’ understanding of the woman’s dependence and attitudes, they provide no support to the woman for an actual quit attempt. Unfortunately, although it is Assisting and Arranging follow-up that are critical to successful quitting [31–33], ,the items in the ‘*Helping’* factor were least likely to be performed (‘Always’ performed by 30 to 45% of survey respondents).

The first factor identified in the analysis of barriers was ‘*Capability*’, which included all the knowledge, skills and confidence (beliefs about capabilities) items. Participants reported poorer knowledge, skills and confidence related to Assisting than Assessing, and more commonly agreed with knowledge statements than with those for skills and confidence – consistent with both their reported behaviour and other research [12, 21, 22]. Importantly, the ‘*Capability’* factor was significantly associated with ‘*Helping’* women, and thus, ascertaining the items in ‘*Capability’* with lesser agreement (skills, confidence and adequate training) helps to identify potential components for interventions. Skills training for health professionals improves professional performance and continuous abstinence [34].

.The *‘Work Environment’* factor was significantly associated with both *‘Helping’* and *‘Assessing quitting’*. There was considerable spread in the responses to the items included in ‘*Work Environment’*, although there was greater agreement with the items related to capacity of the service and pamphlets and resources, than those related to values and social influence. The item with the lowest agreement related to champions within the service, suggesting that developing local cessation champions would be beneficial [35]. Champions may impact the culture of the organisation, motivating other staff, supporting staff training, and monitoring service provision and client outcomes [35].

.The *‘Personal priority’* factor was also associated with *‘Helping’*. There was little disagreement with any of the three items in this factor, however, the item about placing a high priority on smoking cessation was least well supported. Qualitative research has identified that a perceived lack of time to address smoking, combined with a perception that smoking was a ‘private’ issue resulted in smoking cessation being a low priority in some circumstances [12]. One approach to persuading clinicians to prioritise smoking cessation is to ‘reframe’ their perception of smoking by drawing parallels with other conditions and their approach to them [14]. This could involve education about the comparative health consequences of different problems (e.g. smoking vs diabetes), combined with a comparison of the clinical responses and management of each.

The ‘*Intentions and Memory’* factor was also important in predicting *‘Assessing quitting’*. There was stronger agreement with intentions than remembering, with lesser agreement for intentions and memory after the first visit. This suggests that prompts and reminders could be beneficial, particularly focused on subsequent visits – either in the form of enhanced electronic reminders or required fields, or other aids such as flowcharts and clinical pathways displayed in the clinic. As discussed earlier, the high rate of assessment of nicotine dependence identified in this study is likely due to design features of the clinical information system in use.

The final factor significantly related to *‘Assessing quitting’* was *‘Negative perceptions’*. These items were reverse coded, indicating that stronger disagreement with these statements was associated with greater assessment of interest in quitting smoking. It is not surprising that a perception that supporting smoking cessation is difficult and not likely to be successful is associated with lesser assessment of interest in quitting. However, with adequate training, managerial support, provision of useful resources and referral options, this negative perception may be changed.

### Limitations

Despite considerable efforts to maximise response rates, including engaging with the CMC network prior to the survey, using this network to recruit midwives, sending repeated reminders and offering an incentive for participation, the response rate was low. Response rates among health professionals are often low, (25% of American obstetrician-gynecologists [27], and only 6% of Australian physicians [21] responded to similar surveys) and the strategies we implemented are recommended for maximising recruitment [36]. The low response rate is therefore particularly disappointing and the respondents cannot be considered representative of all midwives providing antenatal care in the NSW Health system. However, the objective of this paper was to examine the relationship between perceived barriers and reported provision of support and this internal analysis remains valid as a study’s population does not need to be representative of the population from which it is derived in order to validly ascertain relationships of interest [37–39]. A further limitation is that we relied on self-report of provision of SCS, and there is likely some over-reporting, although the anonymous nature of the survey may have facilitated honest responses. Nonetheless, consistent with other studies, there were many fewer respondents reporting Assisting and Arranging follow-up, suggesting minimal over-reporting.

## Conclusions

In this study, respondents more commonly reported Asking and Assessing than Advising, Assisting, or Arranging follow-up. Three 5As factors were identified– ‘*Helping’*, ‘*Assessing quitting*’ and ‘*Assessing dependence*’. Responses to barrier/enabler items showed greater knowledge, skills, intentions, and confidence with Assessment than Assisting; endorsement for SCS as a priority and part of midwives’ professional role; and gaps in training and organisational support for SCS. Nine barrier/enabler factors were identified. Of these, the factors of ‘*Capability’*; ‘*Work Environment*’ and ‘*Personal priority*’ predicted ‘*Helping’*.

The use of the TDF enabled a systematic and comprehensive approach to identifying barriers to providing SCS, and the multivariate models allowed assessment of the main contributors to poor implementation. This process, including the use of the TDF, facilitates mapping barriers to intervention components using the BCW [14]. Combined with qualitative data already published [12], these results have been mapped to specific, tailored intervention components and behaviour change techniques to form a comprehensive intervention to improve implementation of SCS.

## Supplementary information

**Additional file 1.** is a pdf file containing the questionnaire used in the study.

**Additional file 2 Table S1.** presents the Factor loadings, Cronbach’s alpha and correlations for the factor analysis of provision of the 5As from the Pattern matrix. **Table S2.** presents the data on the frequency of responses to questions on the barriers and enablers to providing the 5As.

## Data Availability

The dataset used during the current study is available from the corresponding author on reasonable request.

## Abbreviations

5As: Ask, Advise, Assess, Assist, Arrange follow-up
BCW: Behaviour change wheel
CMC: Clinical midwifery consultant
LHD: Local health district
SCS: Smoking cessation support
TDF: Theoretical domains framework
