# Supplementary material for: Factors associated with provision of smoking cessation support to pregnant women – a cross-sectional survey of midwives in New South Wales, Australia
Source: BMC Pregnancy Childbirth. 2020 Apr 15;20:219. doi: 10.1186/s12884-020-02912-0 (PMC7161220; doi:10.1186/s12884-020-02912-0)
Supplement: Supplementary file 1 — Additional file 1. is a pdf file containing the questionnaire used in the study. [file 12884_2020_2912_MOESM1_ESM.pdf]

Additional file 1 for article: Factors associated with provision of smoking cessation support to pregnant women – a cross-sectional survey of midwives in New South Wales, Australia. *BMC Pregnancy and Childbirth*. Megan E. Passey, Jo M. Longman, Catherine Adams, Jennifer J Johnston, Jessica Simms, Margaret Rolfe. Corresponding author Megan Passey, University Centre for Rural Health, The University of Sydney, megan.passey@sydney.edu.au

Providing Smoking Cessation Support in Antenatal Care

Survey Tool

Please answer questions in this survey thinking about your current role in public antenatal care

Some questions about you:

Which LHD do you work in? (if more than one then please choose the one where you spend most of your time in antenatal care)

**LHD**	**Please tick ONE**
Central Coast	
Far West	
Hunter New England	
Illawarra Shoalhaven	
Mid North Coast	
Murrumbidgee	
Nepean Blue Mountains	
Northern NSW	
Northern Sydney	
South Eastern Sydney	
Southern NSW	
South Western Sydney	
Sydney	
Western NSW	
Western Sydney	

How many years of midwifery experience have you had? __________ years

What model of antenatal care do you work in? Please tick all that apply and then select the one that you work most in, in antenatal care

**Model of antenatal care**	**Please tick ALL that apply**	**Please tick ONE that is the model of antenatal care you mostly work in**
Team midwifery		
Midwifery group practice (normal risk)		
Midwifery group practice (all risk)		
Midwife and GP Shared Care		
Publically funded homebirth		
Obstetric-led		
Obstetric led for high risk women		
AMIHS		

What is the approximate number of births in the service where you provide most of your antenatal care?

Size of service	Please tick ONE
≤500 births	
501-2,000 births	
2000+ births	

We are interested in your views on providing support for pregnant women to quit smoking.

Please indicate how much you agree or disagree with the following statements

	Strongly agree	Agree	Not sure	Disagree	Strongly disagree	Domain*
I am FAMILIAR with the guidelines for using the 5As for smoking cessation during antenatal care (Ask, Advise, Assess, Assist, Arrange Follow-up)	1	2	3	4	5	Know
I have good KNOWLEDGE of the harms of smoking in pregnancy	1	2	3	4	5	Know
I know how to provide smoking cessation support in antenatal care to help pregnant women quit	1	2	3	4	5	Know
I have good knowledge of nicotine addiction and the barriers to quitting smoking	1	2	3	4	5	Know
I have good knowledge of the use of NRT in pregnancy	1	2	3	4	5	Know
I have the SKILLS required to determine and interpret pregnant women's nicotine dependence	1	2	3	4	5	Skills
I have good skills in MOTIVATING pregnant women who don't want to quit, to try to quit	1	2	3	4	5	Skills
I have good skills in assisting pregnant women with strategies to quit smoking	1	2	3	4	5	Skills
I have good skills in assisting pregnant women who are struggling to quit	1	2	3	4	5	Skills
I've had adequate training in assisting pregnant women to quit smoking	1	2	3	4	5	EC&R
I often find talking with pregnant smokers about their smoking makes me feel uncomfortable	1	2	3	4	5	Emot
I am CONFIDENT assessing women's smoking status	1	2	3	4	5	BA Cap
I am confident providing smoking cessation assistance to pregnant women	1	2	3	4	5	BA Cap
I am confident arranging follow-up support for pregnant smokers	1	2	3	4	5	BA Cap

I INTEND to advise all pregnant smokers to quit	1	2	3	4	5	Int
I intend to provide smoking cessation support to each pregnant smoker	1	2	3	4	5	Int
I intend to follow up with all smokers about their smoking at later visits (after the booking in visit)	1	2	3	4	5	Int
I always REMEMBER to advise women who smoke to quit smoking	1	2	3	4	5	M,A&DP
I always remember to provide smoking cessation support to smoking women at EVERY antenatal visit	1	2	3	4	5	M,A&DP
Most women appreciate it when I discuss quitting smoking with them	1	2	3	4	5	BA Con
I get satisfaction from providing smoking cessation support to pregnant women	1	2	3	4	5	Emot
After the booking in visit, providing smoking cessation support is not as important to me as providing some other aspects of antenatal care	1	2	3	4	5	Goals
I don't have time to provide smoking cessation support in visits after the booking in visit	1	2	3	4	5	EC&R
I feel optimistic that providing smoking cessation support helps women quit smoking	1	2	3	4	5	Opt
Advising women to quit smoking risks pushing them away from antenatal care	1	2	3	4	5	BA Con
I think providing smoking cessation support for pregnant women increases the chances that they'll quit	1	2	3	4	5	BA Con
Helping women quit smoking makes me feel proud of my role	1	2	3	4	5	PR&I
Advising women to quit smoking is one of the main things that can be done to help women have healthy babies	1	2	3	4	5	BA Con
Providing smoking cessation support to women is not worth it given the small level of success	1	2	3	4	5	Opt
Providing smoking cessation support for pregnant women is an important part of my role	1	2	3	4	5	PR&I
Talking with women about quitting smoking is a good use of my time	1	2	3	4	5	BA Con
Referring women to the Quitline is an effective way of assisting pregnant women to quit	1	2	3	4	5	BA Con
The harms of smoking in pregnancy are not as great as the other risks that women face	1	2	3	4	5	BA Con

I place a high priority on helping women quit smoking	1	2	3	4	5	Goals
The team I work with places a high priority on addressing smoking with pregnant women	1	2	3	4	5	Soc Inf
The clinic I work in values midwives who follow the 5As guidelines	1	2	3	4	5	Reinf
I have systems in place (e.g. a checklist or stickers) to help me keep track of women who smoke and provide ongoing smoking cessation support for them	1	2	3	4	5	BR
Our service has systems in place to help keep track of women who smoke and provide ongoing support for them	1	2	3	4	5	EC&R
Our service has good pamphlets and resources to support pregnant smokers to quit	1	2	3	4	5	EC&R
Our service has midwives, obstetricians and/or managers who really champion addressing smoking with our clients	1	2	3	4	5	Soc Inf
Our service has capacity to provide smoking cessation support for pregnant smokers	1	2	3	4	5	EC&R

** The domain name has been added for information but did not appear in the questionnaire. Key to domain names: Know = knowledge; Skills = skills; EC&R = environmental context and resources; Emot = emotions; BA Cap = beliefs about capabilities; Int = intentions; M,A&DP = memory, attention and decision processes; BA Con = beliefs about consequences; Goals = goals; Opt = optimism; PR&I = professional role and identity; Soc Inf = social influences; Reinf = reinforcement; BR = behavioural regulation.*

We are interested in your experience providing support for pregnant women to quit smoking.

Please indicate how frequently you do each of the following

	Always	Usually	Sometimes	Seldom	Never	5A*
How often do you give clear, strong messages urging every pregnant smoker to quit?	1	2	3	4	5	Advise
How often do you identify and document cigarette smoking status for each pregnant patient at the booking in visit?	1	2	3	4	5	Ask
During the booking in visit, how often do you assess whether pregnant women who smoke are willing to try to quit smoking?	1	2	3	4	5	Assess readiness
How often do you assess whether pregnant women who smoke are willing to try to quit smoking at subsequent visits?	1	2	3	4	5	Assess readiness
For women who smoke, how often at the booking in visit do you assess how soon after waking she smokes?	1	2	3	4	5	Assess nicotine dependence

	Always	Usually	Sometimes	Seldom	Never	5A*
For women who smoke, how often at the booking in visit do you assess how many cigarettes she smokes per day?	1	2	3	4	5	Assess nicotine dependence
How often do you assist pregnant women who smoke to quit by discussing barriers to quitting and ways to overcome these?	1	2	3	4	5	Assist
How often do you assist women who are not ready to quit smoking by discussing the benefits of quitting and the risks of continued smoking?	1	2	3	4	5	Assist
How often do you assist pregnant women who smoke to quit by advising on nicotine replacement therapy (NRT)?	1	2	3	4	5	Assist
How often do you provide self-help smoking cessation materials to pregnant women who smoke?	1	2	3	4	5	Assist
How often do you refer pregnant smokers who are willing to quit to the Quitline or other quit smoking services?	1	2	3	4	5	Assist
For pregnant women attempting to quit, how often do you schedule follow-up contact to support their quit attempt?	1	2	3	4	5	Arrange follow-up

* *The 'A' that the item relates to has been added for information but did not appear in the questionnaire.*

Finally, what is your own current smoking status?

**Own smoking status**	**Please tick ONE**
I smoke every day	
I smoke occasionally, but not every day	
I'm an ex-smoker – I never smoke now	
I have never smoked	

THANK YOU we really appreciate the time you have taken to complete this survey.
