# Supplementary material for: Factors associated with provision of smoking cessation support to pregnant women – a cross-sectional survey of midwives in New South Wales, Australia
Source: BMC Pregnancy Childbirth. 2020 Apr 15;20:219. doi: 10.1186/s12884-020-02912-0 (PMC7161220; doi:10.1186/s12884-020-02912-0)
Supplement: Supplementary file 2 — Additional file 2 Table S1. presents the Factor loadings, Cronbach’s alpha and correlations for the factor analysis of provision of the 5As from the Pattern matrix. Table S2. presents the data on the frequency of responses to questions on the barriers and enablers to providing the 5As. [file 12884_2020_2912_MOESM2_ESM.pdf]

Additional file 2 for article: Factors associated with provision of smoking cessation support to pregnant women – a cross-sectional survey of midwives in New South Wales, Australia. *BMC Pregnancy and Childbirth*. Megan E. Passey, Jo M. Longman, Catherine Adams, Jennifer J Johnston, Jessica Simms, Margaret Rolfe. Corresponding author Megan Passey, University Centre for Rural Health, The University of Sydney, megan.passey@sydney.edu.au

Table S1 – Factor loadings, Cronbach's alpha and correlations for factor analysis of provision of the 5As from the Pattern matrix

Factor (Cronbach's alpha)	Item	Loading
Helping (0.862)	How often do you assist pregnant women who smoke to quit by discussing barriers to quitting and ways to overcome these? (*Assist*)	.895
	How often do you assist women who are not ready to quit smoking by discussing the benefits of quitting and the risks of continued smoking? (*Assist*)	.798
	For pregnant women attempting to quit, how often do you schedule follow-up contact to support their quit attempt? (*Arrange follow-up*)	.776
	How often do you provide self-help smoking cessation materials to pregnant women who smoke? (*Assist*)	.653
	How often do you assist pregnant women who smoke to quit by advising on nicotine replacement therapy (NRT)? (*Assist*)	.610
	How often do you give clear, strong messages urging every pregnant smoker to quit? (*Advice*)	.549
	How often do you refer pregnant smokers who are willing to quit to the Quitline or other quit smoking services? (*Assist*)	.459
Assessing Quitting (0.678)	How often do you identify and document cigarette smoking status for each pregnant patient at the booking in visit? (*Ask*)	692
	How often do you assess whether pregnant women who smoke are willing to try to quit smoking at subsequent visits? (*Assess readiness*)	.559
	During the booking in visit how often do you assess whether pregnant women who smoke are willing to try to quit smoking? (*Assess readiness*)	.442
Assessing dependence (0.577)	For women who smoke, how often at the booking in visit do you assess how soon after waking she smokes? (*Assess nicotine dependence*)	.653
	For women who smoke, how often at the booking in visit do you assess how many cigarettes she smokes per day? (*Assess nicotine dependence*)	.652

Kaiser-Meyer-Olkin measure of sampling adequacy 0.877
Bartletts test of sphericity Chi squared=751.6 df=66 p=<0.001
Anti-imaging correlations 0.695-0.926
Helping: Item-to-Total correlations 0.546-0.766; Item-to-item correlations 0.309-0.632
Assessing quitting: Item-to-total correlations 0.404-0.569; Item-to-item correlations 0.324-0.549
Assessing dependence: Item-to-total correlations 0.471; Item-to-item correlations 0.471

Table S2. Frequency of responses to questions on the barriers and enablers to providing 5As (n=150)

Questionnaire Items	Strongly agree %[#]	Agree %	Not sure %	Disagree %	Strongly Disagree %	Original TDF Domain*
**Capability**						
I have good knowledge of the use of NRT in pregnancy	14.7	40.0	25.3	16.0	4.0	Know
I have good skills in assisting pregnant women who are struggling to quit	10.0	37.3	30.7	20.0	2.0	Skills
I have good skills in assisting pregnant women with strategies to quit smoking	12.7	52.7	19.3	14.7	0.7	Skills
I have the skills required to determine and interpret pregnant women's nicotine dependence	15.3	42.7	22.7	15.3	4.0	Skills
I know how to provide smoking cessation support in antenatal care to help pregnant women quit	29.3	54.0	12.0	4.7	0	Know
I have good knowledge of nicotine addiction and the barriers to quitting smoking	30.7	50.0	12.0	7.3	0	Know
I am confident providing smoking cessation assistance to pregnant women	14.7	50.7	22.0	12.0	0.7	BA Cap
I am confident assessing women's smoking status	15.3	66.0	13.3	4.7	0.7	BA Cap
I am confident arranging follow-up support for pregnant smokers	14.0	42.7	27.3	12.7	3.3	BA Cap
I've had adequate training in assisting pregnant women to quit smoking	9.3	32.0	20.7	24.7	13.3	EC&R
I have good skills in motivating pregnant women who don't want to quit, to try to quit	14.0	40.7	31.3	13.3	0.7	Skills
I am familiar with the guidelines for using the 5As for smoking cessation during antenatal care (Ask, Advise, Assess, Assist, Arrange Follow-up)	20.7	42.0	16.0	18.7	2.7	Know
**Intentions and memory**						
I intend to provide smoking cessation support to each pregnant smoker	39.3	53.3	6.0	1.3	0	Int
I intend to follow up with all smokers about their smoking at later visits (after the booking in visit)	30.0	52.7	12.7	3.3	1.3	Int
I always remember to advise women who smoke to quit smoking	34.0	50.0	7.3	8.7	0	M,A&DP
I intend to advise all pregnant smokers to quit	46.7	45.3	6.0	2.0	0	Int
I always remember to provide smoking cessation support to smoking women at every antenatal visit	15.3	38.7	24.0	21.3	0.7	M,A&DP

Questionnaire Items	Strongly agree %#	Agree %	Not sure %	Disagree %	Strongly Disagree %	Original TDF Domain*
**Work environment**						
Our service has good pamphlets and resources to support pregnant smokers to quit	10.7	56.0	16.0	15.3	2.0	EC&R
Our service has capacity to provide smoking cessation support for pregnant smokers	11.3	52.7	17.3	14.0	4.7	EC&R
Our service has midwives, obstetricians and/or managers who really champion addressing smoking with our clients	7.3	30.0	28.0	24.0	10.7	Soc Inf
The clinic I work in values midwives who follow the 5As guidelines	8.7	34.0	42.7	9.3	5.3	Reinf
The team I work with places a high priority on addressing smoking with pregnant women	13.3	42.7	30.0	10.0	4.0	Soc Inf
**Emotional reward**						
Helping women quit smoking makes me feel proud of my role	16.7	64.0	17.3	2.0	0	PR&I
I get satisfaction from providing smoking cessation support to pregnant women	16.7	62.0	17.3	4.0	0	Emot
I think providing smoking cessation support for pregnant women increases the chances that they'll quit	16.0	63.3	17.3	3.3	0	BA Con
I feel optimistic that providing smoking cessation support helps women quit smoking	14.0	46.7	30.0	9.3	0	Opt
Most women appreciate it when I discuss quitting smoking with them	11.3	32.7	37.3	16.0	2.7	BA Con
**Negative perceptions**						
I often find talking with pregnant smokers about their smoking makes me feel uncomfortable	2.7	19.3	7.3	51.3	19.3	Emot
Advising women to quit smoking risks pushing them away from antenatal care	0.7	12.0	22.0	54.0	11.3	BA Con
After the booking in visit, providing smoking cessation support is not as important to me as providing some other aspects of antenatal care	0.7	19.3	14.0	56.7	9.3	Goals
I don't have time to provide smoking cessation support in visits after the booking in visit	6.7	16.7	14.0	55.3	7.3	EC&R
The harms of smoking in pregnancy are not as great as the other risks that women face	0.7	2.7	8.7	64.7	23.3	BA Con
Providing smoking cessation support to women is not worth it given the small level of success	0.7	3.3	7.3	61.3	27.3	Opt
**Personal priority**						

Questionnaire Items	Strongly agree %#	Agree %	Not sure %	Disagree %	Strongly Disagree %	Original TDF Domain*
Providing smoking cessation support for pregnant women is an important part of my role	26.0	66.7	5.3	2.0	0	PR&I
Talking with women about quitting smoking is a good use of my time	23.3	63.3	10.0	3.3	0	BA Con
I place a high priority on helping women quit smoking	19.3	48.7	24.0	8.0	0	Goals
**Tracking systems**						
I have systems in place (e.g. a checklist or stickers) to help me keep track of women who smoke and provide ongoing smoking cessation support for them	6.0	23.3	16.0	38.7	16.0	BR
Our service has systems in place to help keep track of women who smoke and provide ongoing support for them	4.7	24.7	22.7	32.7	15.3	EC&R
**Main things**						
Advising women to quit smoking is one of the main things that can be done to help women have healthy babies	31.3	55.3	8.7	4.7	0	BA Con
**Quitline**						
Referring women to the Quitline is an effective way of assisting pregnant women to quit	16.0	45.3	33.3	4.7	0.7	BA Con
**Not included in factor analysis**						
I have good knowledge of the harms of smoking in pregnancy	59.3	39.3	1.3	0	0	Know

* Note that these items have been grouped under the constructs identified in the factor analysis presented in the paper.
# Percentages may not total 100% due to rounding errors.
Key to domain names: Know = knowledge; Skills = skills; EC&R = environmental context and resources; Emot = emotions; BA Cap = beliefs about capabilities; Int = intentions; M,A&DP = memory, attention and decision processes; BA Con = beliefs about consequences; Goals = goals; Opt = optimism; PR&I = professional role and identity; Soc Inf = social influences; Reinf = reinforcement; BR = behavioural regulation.
